# Mind your step: social cerebellum in interactive navigation

**DOI:** 10.1093/scan/nsac047

**Published:** 2022-07-22

**Authors:** Meijia Li, Min Pu, Kris Baetens, Chris Baeken, Natacha Deroost, Elien Heleven, Frank Van Overwalle

**Affiliations:** Faculty of Psychology and Center for Neuroscience, Vrije Universiteit Brussel, Brussels 1050 , Belgium; Faculty of Psychology and Center for Neuroscience, Vrije Universiteit Brussel, Brussels 1050 , Belgium; Faculty of Psychology and Center for Neuroscience, Vrije Universiteit Brussel, Brussels 1050 , Belgium; Brussels University Consultation Center, Vrije Universiteit Brussel, Brussels 1050, Belgium; Faculty of Medicine and Health Sciences, Department of Head and Skin, Ghent Experimental Psychiatry (GHEP) Lab, Ghent Experimental, Ghent University, Ghent 9000, Belgium; Department of Psychiatry, Vrije Universiteit Brussel, Brussels 1090, Belgium; Department of Electrical Engineering, Eindhoven University of Technology, Eindhoven 5600, The Netherlands; Faculty of Psychology and Center for Neuroscience, Vrije Universiteit Brussel, Brussels 1050 , Belgium; Faculty of Psychology and Center for Neuroscience, Vrije Universiteit Brussel, Brussels 1050 , Belgium; Faculty of Psychology and Center for Neuroscience, Vrije Universiteit Brussel, Brussels 1050 , Belgium

**Keywords:** social navigation, expectation violation, social sequence learning, social interaction, mentalizing, posterior cerebellum, goal-directed behavior, predictive brain

## Abstract

The posterior cerebellum contributes to dynamic social cognition by building representations and predictions about sequences in which social interactions typically take place. However, the extent to which violations of prior social expectations during human interaction activate the cerebellum remains largely unknown. The present study examined inconsistent actions, which violate the expectations of desired goal outcomes, by using a social navigation paradigm in which a protagonist presented a gift to another agent that was liked or not. As an analogous non-social control condition, a pen was transported via an assembly line and filled with ink that matched the pen’s cap or not. Participants (*n* = 25) were required to memorize and subsequently reproduce the sequence of the protagonist’s or pen’s trajectory. As hypothesized, expectation violations in social (*vs* non-social) sequencing were associated with activation in the posterior cerebellum (Crus 1/2) and other cortical mentalizing regions. In contrast, non-social (*vs* social) sequencing recruited cerebellar lobules IV–V, the action observation network and the navigation-related parahippocampal gyrus. There was little effect in comparison with a social non-sequencing control condition, where participants only had to observe the trajectory. The findings provide further evidence of cerebellar involvement in signaling inconsistencies in social outcomes of goal-directed navigation.

## Introduction

Social human interaction is complex and often involves actions that are not always appreciated by others. For example, visiting another person and offering a desired gift is an important but complicated social ritual. Offering an (in)appropriate gift can inform us about the goals and intentions of the giver (Li *et al.*, 2021), and the level of enthusiasm when the gift is accepted can tell us a lot about the preferences of the receiver. Human interaction and protocol prescribe that a gift is accepted with pleasure. But this is often not the case. Gifts are not always appreciated. The purpose of this study is to further investigate whether the cerebellum, which is crucially involved in identifying step-by-step dynamic sequences of human interactions (Heleven *et al.*, 2019, 2021; Cattaneo *et al.*, 2021; Li *et al.*, 2021; Ma *et al.*, 2021; Pu *et al.*, 2021; Van Overwalle *et al.*, 2021), contributes to detecting inconsistent social actions that violate the expectations of desired goal outcomes.

### Cerebral cortex and social cognition

A social ritual, such as gift-giving, requires the understanding of the intentions of gift-givers and preferences of gift-receivers, and more importantly, whether the two match. The ability to infer the mental states of others is termed ‘mentalizing or theory of mind’ (Frith and Frith, 2003; Saxe and Kanwisher, 2003; Atique *et al.*, 2011). It is a human capacity supported by the mentalizing network in the human brain (Gallagher and Frith, 2003; Van Overwalle and Baetens, 2009), which largely overlaps with the ‘default mode network’ (Andrews-Hanna *et al.*, 2014; Spreng and Andrews-Hanna, 2015). Key areas in the mentalizing network are the temporoparietal junction (TPJ) and medial prefrontal cortex (mPFC) in the neocortex, which support social perspective taking (Özdem *et al.*, 2019) and inferring social information such as intentions and personality traits and preferences (Wagner *et al.*, 2016; Heleven and Van Overwalle, 2018; Arbula *et al.*, 2021). Several studies also suggest that these areas show increased activity to prediction errors in social interaction (Saxe *et al.*, 2004; Hampton *et al.*, 2008). When errors occur, observers often wonder ‘why’ this happened, and this triggers mentalizing reasoning in an attempt to understand the error (Spengler *et al.*, 2009; Spunt *et al.*, 2010, 2011; see meta-analysis by Van Overwalle and Baetens, 2009).

Human social goals can also be directly observed from movements, for instance, when navigating through space toward a desired goal. The ‘action observation’ network in social cognition, previously known as the ‘mirror’ network, is responsible for understanding the goal of observed biological movements (see meta-analyses by Van Overwalle and Baetens, 2009; Molenberghs *et al.*, 2012). It is supported by key areas, such as the posterior superior temporal sulcus (pSTS), which detects biological motion (e.g. caressing *vs* punching), the anterior intraparietal sulcus (aIPS), which puts the movement in a context of an object (e.g. caressing a close *vs* distant other) and lastly the premotor cortex (PMC), which supports the goal-directed action observation underlying the movement (e.g. soothing *vs* hurting someone).

### Cerebellum and social sequencing

In addition to the neocortex, recent research points to the essential role of the cerebellum in identifying and learning dynamic sequences in social actions, leading to automatized rituals in social interaction. This idea originated from the ‘sequencing hypothesis’ put forward by Leggio *et al.* (2011) and Leggio and Molinari (2015), which suggests that the cerebellum is involved in ‘sequencing incoming sensory patterns and outgoing responses’ and so governs motor and non-motor coordination and planning. For example, an important function of the cerebellum is to automatize motor sequences, such as walking and riding a bike or a car (Leggio and Molinari, 2015; Van Overwalle *et al.*, 2019b, 2020a). Research has demonstrated that the posterior cerebellum, especially lobules Crus 1 and 2, is implicated in social sequence learning (Van Overwalle *et al.*, 2014; Li *et al.*, 2021) and is consistently recruited when sequences of social actions are identified or memorized in a variety of tasks involving both implicit and explicit social learning (Heleven *et al.*, 2019, 2021; Cattaneo *et al.*, 2021; Ma *et al.*, 2021; Pu *et al.*, 2021; Van Overwalle *et al.*, 2021).

### Cerebellum and social expectation violation

An important implication of the sequencing hypothesis of the cerebellum is that this area is not only essential for the identification and learning of sequences but also for detecting and correcting errors in sequences (Leggio and Molinari, 2015; Popa *et al.*, 2016). Interestingly, recent evidence suggests that the posterior cerebellum not only responds to sequence violations but also to errors at a higher cognitive level. For example, Moberget *et al.* (2014) found that the posterior cerebellum was involved in the language domain when semantic predictions were violated. More importantly, Pu *et al.* (2021) demonstrated that the posterior cerebellum responded to violations in social cognition, such as the implied trait of actions, for example, when an honest person starts telling lies.

### Cerebellum and social–spatial navigation

In exploring the mechanisms of human spatial navigation, meta-analyses identified the hippocampus and the adjacent middle temporal areas [e.g. parahippocampal gyrus (PHG)] as the primordial supporting subcortical sites (Kühn and Gallinat, 2014; Kong *et al.*, 2017; Qiu *et al.*, 2019). Spatial navigation is further supported by cortical areas, including the precuneus (PCun) involved in encoding visual landmarks as part of the external scene and the supplementary motor area (SMA) involved in identifying visual landmarks for navigational goals (Qiu *et al.*, 2019).

Human social interaction also comprises navigating in a spatial environment where we physically meet other people (Proulx *et al.*, 2016), which is a basic human need. However, unlike ‘spatial’ navigation that focuses on the navigation of the self through space and locations, ‘social interactive navigation’ (Tavares *et al.*, 2015; Li *et al.*, 2021) refers to observing how other people navigate and interact with each other in a socially rich environment and thus heavily relies on the capacity for social reasoning and cognition. Evidence indicates that social cognition shares some underlying brain mechanisms with spatial navigation (Table 1; Proulx *et al.*, 2016; Schafer and Schiller, 2018; Spreng *et al.*, 2009). First, key areas of the ‘action observation’ network in social cognition, including the PMC and aIPS, are located close to areas in the posterior-dorsal module of the navigation network (Kong *et al.*, 2017), suggesting a common process of observation of biological movement. Second, the PCun is activated in both social cognition and spatial navigation, indicating that it provides a spatial setting for social actions for social mentalizing (Costigan *et al.*, 2019) as well as visual landmarks in spatial navigation (Qiu *et al.*, 2019). A recent study using spatial trajectories traversed by human-like agents by Li *et al.* (2021) emphasizes the combined recruitment of social and spatial brain areas. The study found cerebellar Crus 1 recruitment in social compared to non-social trajectories, paralleled by strong activation in cortical key regions of the mentalizing network (e.g. TPJ) and of the action observation network (e.g. pSTS, aIPS and PMC) as well as in the related SMA (Mukamel *et al.*, 2010; Acharya and Shukla, 2012; Aridan and Mukamel, 2016).

**Table 1.** ROIs derived from meta-analyses in social cognition and spatial navigation

	Coordinates		
ROIs	*x*	*y*	*z*
Social			
Action sequencing			
Cerebellar Crus 2	±25	−75	−40
Cerebellar Crus 1	±40	−70	−40
Mentalizing			
mPFC	0	50	20
TPJ	±50	−55	25
PCun*	0	−60	40
Goal-directed action observation			
PMC*	±40	5	40
aIPS*	±40	−40	45
pSTS	±50	−55	10
Spatial			
Memory (medial-temporal module)			
L PHG	−25	−36	−13
R PHG	26	−34	−13
Landmark identification (anterior module)			
L SMA	−4	12	51
R SMA	6	12	52

*Note:* For social cognition, we included all areas identified in meta-analyses on the cortex (Van Overwalle, 2009; Van Overwalle and Baetens, 2009) and the cerebellum (Van Overwalle *et al.*, 2020a). For spatial navigation, we included all areas from the modules identified by Kong *et al.* (2017), Kühn and Gallinat (2014) and Qiu *et al.* (2019), excluding those that overlap with social cognition, which were denoted by *(identified as PCun, precentral gyrus and angular gyrus, respectively). Occipital/Visual areas were omitted. Adapted from Li *et al.* (2021). The MNI coordinates came from these meta-analyses and were used as centers for our ROI analysis.

Spatial and social navigation may share other similarities, including visuo-spatial working memory. This process is closely involved in navigation and includes a variety of different cognitive functions, such as route learning (Garden *et al.*, 2002) and configural learning (Münzer *et al.*, 2012). However, in the present design, these processes were largely controlled by keeping them identical between human social and non-human sequence learning.

### Present study

**Fig. 1.**
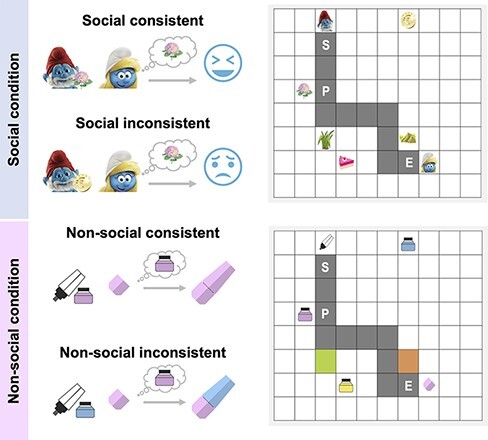
Experimental design. The task involved an 8 × 10 grid. Shown in gray (not visible to the participants) are trajectories from the Social and Non-social conditions. We explain the task here with the aid of an actual trial. Before each trial, participants were informed of the gift preference of Smurfette (prefers flowers) or the ink the pen should be filled with (pen should be filled with ink whose color is consistent with the cape at the end). In the Social condition, Papa Smurf will pass by the gift and pick it up. In the consistent condition, the gift (flower) is desired by Smurfette as indicated here by , whereas in the inconsistent condition, the gift (coin; not shown on the grid) is not desired as indicated here by . In the Non-social conditions, the pen is filled with ink whose color is consistent/inconsistent with the cap at the end. In each trajectory, the smurf/pen moved from the starting point to an endpoint at a fixed pace (400 ms per step). In the Sequencing conditions, participants were instructed to observe and memorize the trajectories carefully (Observation Phase) and then to reproduce the same movements with direction buttons (Reproduction Phase). In the Social Non-sequencing conditions, everything was similar, except that the Reproduction Phase was skipped. Note that the following symbols were not shown to the participants: S, starting point; P, gift pick-up/ink fill-in point; E, endpoint; thought balloons, emoticons.

The present study aimed to investigate whether the cerebellum detects social expectation violation in social navigation. To do so, we adopted a social interactive navigation paradigm modified from the previous study by Li *et al.* (2021), depicting human-like agents moving through a grid-like space during social interaction. To ensure that navigation was socially driven and goal-directed, we made the present task interactive by including two agents involved in gift offering and receiving. In the main experimental Social Sequencing condition (Figure 1), participants observed and memorized the movements in a trajectory of an agent picking up a gift and walking toward the recipient who either liked the gift (Consistent with the receiver’s preference) or not (Inconsistent). Then, they were required to reproduce the trajectories (Sequencing) or not required to reproduce them (Non-sequencing control). As Non-social control, we created similar trajectories described as a factory production line, where highlighters were filled with ink whose color was either identical to the cap (Consistent with the pen cap’s color) or not (Inconsistent).

Based on the sequencing hypothesis in social cognition (Van Overwalle *et al.*, 2020a) and previous work (Li *et al.*, 2021), we hypothesize that social trajectories would elicit more activation in the posterior cerebellum than non-social control trajectories or non-sequential conditions (i.e. when the trajectory does not have to be learned and reproduced). Other cortical mentalizing areas would be responsive to social *vs* non-social scenarios, but much less to sequencing because the cerebellum is expected to be more sensitive to sequencing. Importantly, our novel ‘social violation hypothesis’ is that the posterior cerebellum and cortical mentalizing areas will be additionally responsive to violations of social expectations, in this case, when social interaction leads to unexpected and undesired effects (i.e. the wrong gift). Given the error manipulation in the present experiment, the action observation network would probably play a diminished role, as social errors tend to recruit predominantly the mentalizing network as argued earlier.

In line with the sequencing hypothesis, which points to the role of the cerebellum in both sequence detection and generation, we hypothesized that the predicted effects of sequencing would be observed during both the observation and the reproduction of trajectories.

## Method

### Participants

Data were from 25 right-handed, native Dutch-speaking individuals (male = 7) with age varying from 20 to 32 years (*M *= 23.96, s.d. = 3.44). The sample size was determined based on earlier work on social action sequences or spatial tasks, which usually use a sample size of around 25, such as Haihambo *et al.* (2021; social action sequence, *N* = 27), Heleven *et al.* (2019; social action sequence, *N* = 24), Pu *et al.* (2021; social action sequence, *N* = 26), Rachman *et al.* (2019; social voice sequence, *N* = 25) and Gilbert *et al.* (2007; spatial task, *N* = 16). They all reported no abnormal neurological history and had normal or corrected-to-normal vision. All participants were right-handed, as assessed by the Dutch version of the Edinburgh Inventory (Oldfield, 1971). An additional participant could not finish the study due to a headache in the scanner and was not included. Informed consent was obtained with the approval of the Medical Ethics Committee at the Hospital of the University of Ghent, where the study was conducted. Participants were paid 20 euros for their participation and additional reimbursement for public transportation costs.

### Experimental task

The experiment involved a within-participant design with the following three factors: Domain (Social *vs* Non-social), Consistency (Consistent *vs* Inconsistent) and Task (Sequencing *vs* Non-sequencing). Because of time constraints (i.e. to complete the task in the scanner in 45 min), we did not include Non-social Non-sequencing conditions, leaving six conditions overall.

The main condition was the Social Sequencing condition (Figure 1). In each trial, participants saw an 8 × 10 grid taking 71% of the width and 80% of the height of the screen. In the grid, a protagonist (one out of six smurfs, well-known Belgian cartoon figures) was moving through the grid, populated with some desirable objects and obstacles (e.g. flower gifts and rock obstacles). There were no actual (biological) movements, but quick shifts of the head of the smurf from one cell in the grid to an adjacent cell. Participants received the following instructions: ‘Smurfs are social beings, and typically want to make others happy. They visit each other and bring with them a gift to the other smurf to make him/her happy. … Every smurf loves one item and is always happy when he/she receives it as a gift’. Participants were then told that ‘when an object transferred to the smurf when he/she was passing by, this indicated that the object was taken and carried as a gift’. Finally, they were instructed, ‘First, look carefully at the correct steps in the trajectory that a smurf takes. Afterward, repeat these steps’. To create a robust expectation in the participant, the same desired gift was shown at the beginning of each trial before the protagonist started moving. In the Consistent condition, the protagonist picked up the gift desired by the receiver, whereas in the Inconsistent condition, the protagonist picked up a gift not desired by the receiver. Since participants were informed in advance what the desired gift was (see section ‘Procedure’), they could infer at this point onward whether the receiving smurf would (not) like the gift. To explain why the movements of the smurfs were sometimes erratic and not directed straightforward toward the gift, they were additionally told: ‘You will see different trajectories in which, in addition to these objects, some obstacles (stones, tree stump or grass) obstruct the view and the passage of the smurfs. The smurfs can only see a few steps ahead and must therefore get close to the objects to see them clearly’.

In Non-social Sequencing control condition (Figure 1), the six giving smurfs, six gifts and six receiving smurfs were replaced by six pens, six ink tanks and six pen caps, respectively. On each trial, the grid represented an assembly line of highlighters. Participants were given the following instructions: ‘In a highlighter factory production line, the highlighters need to be filled with ink, then the pen will be covered with its corresponding cap at the end before passing the inspection’. When the pen was passed by the ink tank, the pen was automatically filled with colored ink. In the Consistent condition, the pen was filled with ink whose color was identical to the cap at the end, whereas in the Inconsistent condition, the pen was filled with a different color. Since participants could see the color of the ink and the cap, they could infer at this point onward whether the ink and cap were consistent or not. Note that the inconsistency in the non-social assembly line suggests a mechanical error, whereas the inconsistency in the social gift scenario suggests oversight or crudeness, which are social mentalizing errors.

To identify the critical role of sequencing, we also created a Social Non-sequencing control condition with both consistent and inconsistent trials, in which the participants were instructed to passively observe the movement of the smurfs without memorizing or reproducing the trajectory. All other aspects of the task and material were identical to the Sequencing conditions, including the requirement to memorize the gift preference of the receiving smurf.

### Stimulus material

We initially created 24 pairs of trajectories, of which 12 pairs of trajectories were easy (10 steps, 2–3 turns) and 12 pairs were hard (14 steps, 4–6 turns) to generate sufficient variation in performance (see also Pu *et al.*, 2020). To pilot test and select the material, 30 participants (age: *M *= 19, s.d. = 2.46, range 19–30, male = 1) rated to what extent the trajectory of the agent and the positions of the obstacles were reasonable on a five-point scale (1 = not reasonable, 5 = very reasonable) and their preference for one of the two trajectories (1 = trajectory one, 2 = trajectory two). For the experiment, we selected 10 easy pairs and 10 hard pairs with the highest reasonability ratings and no preference for one of the two trajectories. The reasonability scores for the selected 20 pairs of trajectories were all beyond 2.4 on the five-point scale (Easy: *M ± *s.d. = 3.41 ± 0.43; Hard: *M ± *s.d. = 3.26 ± 0.48). We then generated three additional equivalent sets of trajectories by mirroring the selected trajectories upside–down, left–right or both. From those trajectories, we randomly selected 108 trials in total and 36 trials for each condition.

### Procedure

Before the participants entered the scanner, they were familiarized with the task and the direction buttons for moving through the grid using the keyboard by practicing 20 easy version non-social trajectories (which were not part of the experimental stimulus material). Feedback on their average accuracy in reproducing the trajectories was given after every five trials. They could enter the scanner once they had achieved 90% accuracy or higher. When participants were in the scanner, they also practiced five trials to get familiar with performing the task and the response box inside the scanner. Then, the task began.

Every participant finished three conditions (Social Sequencing, Non-social Sequencing and Social Non-sequencing control), where each condition involved 36 trials, including 60% consistent and 40% inconsistent trials presented in random order.

Participants first finished the Non-social Sequencing condition and then took the Social Sequencing condition followed by the Social Non-sequencing condition. The Non-social Sequencing condition was presented first to avoid possible leaking from social to non-social conditions, that is, to rule out that the non-social pen and ink would be anthropomorphized (e.g. Lockwood *et al.*, 2020) like in the often-used Heider–Simmel test where triangles are often understood as moving in a human-like manner (cf., Van Overwalle and Baetens, 2009), whereas the Non-sequencing condition was presented last, because it required no memorization so that performance would be less influenced by fatigue effects.

In the Sequencing condition, each trial comprised an Observation and a Reproduction phase. Before each trial, participants were informed of the gift preference of the receiving smurf (e.g. prefers flowers) or the ink the pen should be filled with (pink cap should be filled with pink ink). In the Observation Phase, each trial began with a blank screen with a duration jittered randomly from 0 to 1000 ms. After a warning signal ‘Look carefully’ presented for 3000 ms on the screen, the smurf/pen began to move at a fixed pace of 400 ms per step toward a gift/ink tank and to the other smurf/cap. When the smurf passed by the gift, the smurf picked it up and took it with him for the rest of the trajectory. Likewise, when the empty pen passed by the ink tank, the pen was filled with the ink and this remained so till the end of the trajectory. Each trajectory started at any possible cell in the grid, except for the bordering and central cells of the grid. Participants had to observe and memorize the trajectories produced by the smurf/pen.

Afterward, the Reproduction Phase started. Participants had to reproduce the trajectory movements in the grid using button presses at their own pace (1 = up, 2 = down, 3 = left and 4 = right). If they made a mistake at one step, an error warning appeared on the screen for 1000 ms, after which they could continue their movements from the place before the mistake occurred.

After reproducing the trajectory, participants were asked two questions (while smurfs and gifts/pens and ink were represented by their respective pictures used in the grid) as a manipulation check and to ensure that they paid attention to the consistency of the gift/ink. The Gift/Ink question asked: ‘What gift did this smurf receive?’/‘Which ink was the pen filled with?’ and the Outcome question asked: ‘Was this smurf happy with this gift (yes/no)?’/‘Was the ink color correct (yes/no)?’ with 5 s to answer.

In the Social Non-sequencing conditions, the participants completed only the Observation Phase and answered the two questions immediately after each trial.

### Questionnaires

#### Autism questionnaire

To ensure that all participants had no Autism spectrum disorder (ASD) traits, we administered the ‘social responsiveness scale for adults’ (SRS-A). The SRS-A contains 65 questions using a four-point rating scale (1 = never, 2 = sometimes, 3 = often and 4 = almost always), assessing five different areas: social awareness, social cognition, social communication, social motivation and autistic mannerisms. A cut-point of 70 for males and 65 for females of SRS total raw score is recommended to screen for autism spectrum conditions in the general population group (Constantino and Gruber, 2012). In our sample, the mean score was 45.50 for males (range between 22 and 69) and 37.29 for females (range between 11 and 54). All scores were well below the clinical threshold.

### Functional magnetic resonance imaging (fMRI) data acquisition

Images were collected with a Siemens Magnetom Prisma fit 3 T scanner system (Siemens Medical Systems, Erlangen, Germany) using a 64-channel radiofrequency head coil. Stimuli were projected onto a screen at the end of the magnet bore, and the participant viewed the stimuli through a mirror mounted on the head coil. Stimulus presentation was controlled by E-Prime 3.0 (www.pstnet.com/eprime; Psychology Software Tools) under Windows XP. Participants were placed headfirst and supine in the scanner bore and were instructed not to move their heads to avoid motion artifacts. Foam cushions were placed within the head coil to minimize head movements. First, high-resolution anatomical images were acquired using a T1-weighted 3D magnetization-prepared rapid gradient echo (MPRAGE) sequence (repetition time (TR) = 2250 ms, echo time (TE) = 4.18 ms, inversion time (TI) = 900 ms, field of view (FOV) = 256 mm, flip angle = 9º, voxel size = 1 mm  × 1 mm  × 1 mm). Second, a field map was calculated to correct inhomogeneities in the magnetic field (Cusack and Papadakis, 2002). Third, whole-brain functional images were collected in a single run using a T2*-weighted gradient echo sequence, sensitive to BOLD contrast (TR = 1000 ms, TE = 31.0 ms, FOV = 210 mm, flip angle = 52º, slice thickness = 2.5 mm, distance factor = 0%, voxel size = 2.5 mm  × 2.5 mm × 2.5 mm, 56 axial slices, acceleration factor generalized autocalibrating partially parallel acquisitions (GRAPPA) = 4).

### fMRI data preprocessing

SPM12 (Wellcome Department of Cognitive Neurology, London, UK) was used to process and analyze the fMRI data. To remove sources of noise and artifacts, data were preprocessed. Inhomogeneities in the magnetic field were corrected using the field map (Cusack and Papadakis, 2002). Functional data were corrected for differences in acquisition time between slices for each whole-brain volume, realigned to correct head movement and co-registered with each participant’s anatomical data. Then, the functional data were transformed into standard anatomical space (2 mm isotropic voxels) based on the ICBM152 brain template [Montreal Neurological Institute (MNI)]. Normalized data were then spatially smoothed (6-mm full-width at half-maximum) using a Gaussian Kernel. Finally, we used the Artifact Detection Tools (https://web.mit.edu/swg/art/art.pdf; https://www.nitrc.org/projects/artifact_detect) to examine the preprocessed data for excessive motion artifacts and correlations between motion and experimental design and between global mean signal and experimental design. Outliers were identified in the temporal differences series by assessing between-scan differences (*Z*-threshold: 3.0 mm, scan to scan movement threshold: 0.5 mm; rotation threshold: 0.02 radians). These outliers were omitted from the analysis by including a single regressor for each outlier. A default high-pass filter was used for 128 s, and serial correlations were accounted for by the default auto-regressive AR(1) model.

### fMRI data analysis

The general linear model of SPM12 (Wellcome Department of Cognitive Neurology, London, UK) was used to conduct the analysis of the fMRI data. At the first (single participant) level, the event-related design was modeled based on all six conditions, involving Domain (Social *vs* Non-social) × Consistency (Consistent *vs* Inconsistent) × Task (Sequencing *vs* Non-sequencing) as within-participant factors. We created two models for the analysis, because our design was not fully factorial and lacked a Non-Social Non-sequencing condition. First, to test the main hypothesis on a Social *vs* Non-social contrast, we built a first model involving four regressors reflecting the four cells from the two factors Domain (Social *vs* Non-social) × Consistency (Consistent *vs* Inconsistent) and applying these regressors in each of the two phases (Observation and Reproduction), resulting in eight regressors overall. Second, to test the Sequencing hypothesis on a Sequencing *vs* Non-sequencing contrast, we built a second model involving all three conditions in the Observation Phase (Social Sequencing, Non-social Sequencing and Social Non-sequencing) × Consistency (Consistent *vs* Inconsistent), resulting in six separate regressors overall.

During the Observation and Reproduction Phase, onsets were specified at the Gift pick-up/Ink fill-in time point since the expectation of the participants will be met or violated at that given timepoint of the trajectory. Duration was set to zero to focus the analysis on the start of the violation. Each trial onset was convolved with a canonical hemodynamic response function and its dispersion and temporal derivatives. During the Reproduction Phase, only onsets of correct steps were included in the analysis. The number of errors was not introduced as an individual covariate in the analysis since the error rate was generally quite low (<6%).

At the second (group) level, clusters from the whole-brain analysis were defined at threshold *P* < 0.001, uncorrected with a minimum cluster extent of 10 voxels, and we report the results of clusters with a family wise error (FWE)-corrected cluster-wise threshold at *P *< 0.05. For all phases and all questions, we conducted a within-participant one-way analysis of variance (ANOVA) and defined all possible *t*-contrasts between regressors of interest (see the ‘Results’ section). A surface-based flatmap representation of significant peaks in the cerebellum with *P* < 0.001 (uncorrected) was made using the spatially unbiased infratentorial template (SUIT, Diedrichsen, 2006). The functional threshold was adjusted to correspond to a cluster-wise *P* < 0.05 FWE-corrected threshold from the second-level analysis (Diedrichsen and Zotow, 2015). This led to an adjusted threshold of *t *= 3.14.

### Regions of interest (ROIs)

**Fig. 2.**
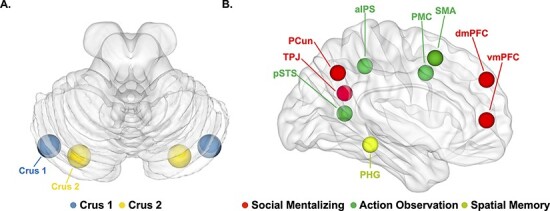
ROIs utilized in the fMRI analysis. The regions were selected from previous research. (A) Posterior cerebellar Crus 1 and 2. (B) ROIs in the left hemisphere. Image generated using BrainNet Viewer software (Xia *et al.*, 2013).

To explore our hypothesis in depth, ROIs for social cognition and spatial navigation were specified and analyzed (Li *et al.*, 2021; see Table 1 for MNI coordinates and Figure 2 for locations). For the posterior cerebellum, ROIs were centered around the MNI coordinates of Crus 1 and 2, taken from earlier Dynamic causal modeling (DCM) studies on social sequencing (Van Overwalle *et al.*, 2019c, 2020b). The cerebral ROIs involved in mentalizing and actin observation were identified from the meta-analysis by Van Overwalle (2009) and Van Overwalle and Baetens (2009) and additionally involved the bilateral SMA and the bilateral PHG. The ROIs were used to perform a small volume correction using the same thresholds as the whole-brain analysis. To accommodate for the volume differences of distinct brain parts and avoid substantial overlap between cerebellar ROIs, we used spheres with a radius of 5 mm for cerebellar ROIs (Crus 1 and 2) and 10 mm for all other neocortical ROIs (see also Van Overwalle *et al.*, 2019c).

### Behavioral data analysis

For the Sequencing condition, accuracy in the Reproduction Phase was calculated for each trial separately by dividing the number of correct steps by the total steps for each trajectory. Reaction time (RT) to reproduce the whole trajectories was also recorded as well as the accuracy of the manipulation check questions (Gift/Ink question and Outcome question).

Data were analyzed with repeated-measures ANOVAs, with Domain (Social *vs* Non-social) and Consistency (Consistent *vs* Inconsistent) as within-participant factors in Sequencing conditions. A Greenhouse–Geisser correction was used if sphericity was not assumed. Partial eta squared was calculated as a measure of effect size. The data were analyzed with SPSS 25.0.

## Results

### Behavioral results

#### Reproduction of trajectory

Accuracy and reaction time (RT) were analyzed using a repeated-measures ANOVA with Domain (Social *vs* Non-social) × Consistency (Consistent *vs* Inconsistent) as within-participant factors (Table 2). As for the RT, we found that the Domain main effect was significant [*F* (1, 24) = 32.72, *P *< 0.001, η_p_^2^ = 0.58, 1–β = 1.00]. Reproducing social trajectories was quicker compared to the Non-Social trajectories (*Mean difference (MD)* = 1.04). No other main effect or interactions on accuracy or RT was significant.

**Table 2.** Descriptive statistics of behavioral data (means ± s.d.)

		Social Sequencing	Non-social Sequencing	Social Non-Sequencing
Consistent	Reproduction of trajectory			
	Accuracy (%)	95.27 ± 3.21	95.31 ± 2.59	
	RT (s)	9.75 ± 2.22	10.79 ± 2.40	
	Gift/Ink question			
	Accuracy (%)	97.73 ± 4.61	98.65 ± 2.57	98.64 ± 2.24
	Outcome question			
	Accuracy (%)	97.73 ± 4.54	97.89 ± 3.51	97.63 ± 3.26
Inconsistent	Reproduction of trajectory			
	Accuracy (%)	94.65 ± 3.07	95.6 ± 3.18	
	RT (s)	9.75 ± 1.74	10.79 ± 2.61	
	Gift/Ink question			
	Accuracy (%)	90.99 ± 9.50	88.51 ± 15.48	94.53 ± 6.41
	Outcome question			
	Accuracy (%)	94.16 ± 6.69	85.37 ± 23.86	93.26 ± 7.89

*Note*: Reproduction accuracy = number of correct steps divided by the total steps for each trajectory. Reproduction RT = time for reproducing the whole trajectory. No reproduction task was included in the Social Non-sequencing condition.

#### Manipulation check

Accuracies in the Gift/Ink question and Outcome question were analyzed using a repeated-measures ANOVA with Domain (Social *vs* Non-social) × Consistency (Consistent *vs* Inconsistent) as within-participant factors (Table 2). We found that the Consistency main effect was significant for both questions [Gift/Ink question: *F* (1, 24) = 16.92, *P *< 0.001, η_p_^2^ = 0.41, 1 – β = 0.98; Outcome question: *F* (1, 24) = 7.84, *P *< 0.01, η_p_^2^ = 0.25, 1 – β = 0.77]. In the consistent condition, the accuracy was generally higher than in the inconsistent condition (*MD*_Gift/Ink_ = 8.44%; *MD*_Outcome_ = 8.05%). However, all accuracies were higher than 85%. No other main effect or interactions was significant.

In summary, reproducing social trajectories was easier than non-social trajectories. Also, inconsistencies resulted in lower accuracies on the manipulation check questions.

### fMRI results

To recall, we hypothesized that social trajectories would elicit more activation in the posterior cerebellum than non-social control trajectories or non-sequential conditions. Other cortical mentalizing areas would be responsive to social *vs* non-social scenarios, but much less to sequencing, because only the cerebellum would be preferentially sensitive to sequencing. Importantly, our novel social violation hypothesis states that the posterior cerebellum and cortical mentalizing areas will be additionally responsive to social violations (i.e. wrong gift) and less so to non-social violations (i.e. wrong ink). Therefore, we will compute the comparisons separately for the inconsistent and consistent conditions.

Note that for all these contrasts of interest, we also computed the opposite contrast as a control. To avoid redundancy, we first report the results of the ROI analysis using small volume correction and then additional clusters of the whole-brain analysis (Tables 3–5) for each contrast. Coordinates of the ROIs are listed in Table 1, and the ROI results are summarized in Table 6. For ease of presentation, we treat the SMA as an extended part of the action observation network (Table 6; see also Ertelt *et al.*, 2007).

**Table 3.** Whole-brain and ROI analysis during the observation of the trajectories

	MNI coordinate				
Contrasts and anatomical label	*x*	*y*	*z*	Voxels	*max t*
Inconsistent: Social Sequencing > Non-social Sequencing					
R cerebellum (Crus 2), including Crus 1°	36	−76	−38	287	4.54
R cerebellum (Crus 2)	22	−86	−36		3.88
R rectal gyrus	2	36	−16	762	5.05*
R medial frontal pole, including vmPFC°	2	58	0		4.57
L medial orbital gyrus	−8	36	−12		4.42
L middle frontal gyrus	−24	24	44	302	4.77
L paracentral lobule	−4	−28	64	972	6.40^***^
R PCun	6	−48	54		6.05^***^
L PCun	−14	−56	60		4.7
R middle temporal gyrus	44	−66	2	2332	9.03^***^
R middle temporal gyrus, including pSTS°	56	−64	12		7.22^***^
R superior temporal gyrus, including TPJ°	56	−44	16		6.96^***^
L middle temporal gyrus, including pSTS°	−42	−66	6	1380	6.86^***^
L angular gyrus	−48	−62	46		4.61
L inferior parietal lobule, including TPJ°	−50	−74	26		4.59
R cuneus	18	−98	10	272	6.61^***^
Inconsistent: Non-social Sequencing > Social Sequencing					
R precentral gyrus, including aIPS°	36	−24	58	51 165	21.36^***^
L cerebellum (IV–V)	−20	−50	−24		21.03^***^
R precentral gyrus	42	−20	62		18.51^***^
R middle frontal gyrus	30	40	30	890	5.23*
R middle frontal gyrus	28	52	26		4.94*
R middle frontal gyrus	40	38	30		4.6
L precentral gyrus	−32	−14	64	281	7.39^***^
Consistent: Social Sequencing > Non-social Sequencing					
R PCun	6	−50	54	670	6.58^***^
R superior parietal lobule	16	−58	62		4.67
L PCun	−12	−58	60		4.47
R middle temporal gyrus, including pSTS°	42	−64	12	1902	7.51^***^
R middle temporal gyrus	42	−64	2		6.95^***^
R middle temporal gyrus	54	−68	0		6.28^***^
L middle temporal gyrus	−42	−68	8	769	5.63^**^
L inferior parietal lobule	−48	−76	28		4.32
L middle temporal gyrus, including pSTS°	−50	−58	10		4.25
ROI: R TPJ	50	−46	20	71	4.88^***^
ROI: L TPJ	−48	−54	18	24	3.90^**^
Consistent: Non-social Sequencing > Social Sequencing					
R precentral gyrus	36	−24	58	61 533	20.08^***^
L cerebellum (IV–V)	−18	−50	−24		19.39^***^
R precentral gyrus, including aIPS°	40	−16	54		17.27^***^
L precentral gyrus	−34	−14	64	320	6.82^***^

*Note*: Coordinates refer to the MNI stereotaxic space. Whole-brain and ROI analysis threshold at voxel-wise uncorrected *P* < 0.001 with voxel extent ≥ 10, with cluster-wise FWE corrected *P* < 0.05. Only the highest peaks of each cluster are shown, except for the cerebellum and significant ROIs. L = left, R = right.

* *P* < 0.05,

** *P* < 0.01,

*** *P* < 0.001 (peak FWE corrected) after whole-brain analysis. °*P* < 0.001 cluster-level FWE corrected using a small volume correction of a sphere with a 5- or 10-mm radius and centered around a priori MNI coordinate. These results are also summarized in Table 6.

**Table 4.** Whole-brain and ROI analysis during the observation of the trajectories for Social Sequencing and Social Non-sequencing control contrasts

	MNI coordinate				
Contrasts and anatomical label	*x*	*y*	*z*	Voxels	max *t*
Inconsistent: Social Sequencing > Social Non-Sequencing					
L PCun	−26	−52	14	4164	7.98^***^
R hippocampus	36	−40	−2		7.57^***^
R PCun	26	−44	16		7.32^***^
Inconsistent: Social Non-Sequencing > Social Sequencing					
R Precentral gyrus	34	−22	56	54 814	15.42^***^
L cerebellum (VI)	−26	−52	−22		13.87^***^
R posterior-medial frontal, including SMA°	6	−4	58		13.46^***^
L middle temporal gyrus	−50	−24	−4	175	4.45
Consistent: Social Sequencing > Social Non-Sequencing					
L PCun	−24	−48	16	4039	7.85^***^
R PCun	26	−46	14		7.33^***^
Thalamus: temporal	−16	−26	26		6.73^***^
Consistent: Social Non-Sequencing > Social Sequencing					
R precentral gyrus	34	−24	58	60 851	14.57^***^
L cerebellum (VI)	−26	−54	−22		13.7^***^
R precentral gyrus	34	−20	68		12.74^***^
R IFG (p. triangularis)	46	24	30	560	5.56^**^
R IFG (p. opercularis), including PMC°	34	10	30		4.72
R superior temporal gyrus	46	−30	−2	218	4.77

*Note*: Coordinates refer to the MNI stereotaxic space. Whole-brain and ROI analysis threshold at voxel-wise uncorrected *P* < 0.001 with voxel extent > 10, with cluster-wise FWE corrected *P* < 0.05. Only the highest peaks of each cluster are shown, except for the cerebellum and significant ROIs. L = left, R = right.

* *P* < 0.05,

** *P* < 0.01,

*** *P* < 0.001 (peak FWE corrected) after whole-brain analysis. °*P* < 0.001 cluster-level FWE corrected using a small volume correction of a sphere with a 5- or 10-mm radius and centered around a priori MNI coordinate. These results are also summarized in Table 6.

**Table 5.** Whole-brain and ROI analysis during the reproduction of the trajectories

	MNI coordinate				
Contrasts and anatomical label	*x*	*y*	*z*	Voxels	*max t*
Inconsistent: Social Sequencing > Non-social Sequencing					
R cerebellum (Crus 2)	36	−76	−40	539	5.04*
R cerebellum (Crus 2)	24	−86	−36		4.68
R cerebellum (Crus 1)	24	−80	−30		3.99
L cerebellum (Crus 2)	−14	−80	−44	201	4.67
L cerebellum (Crus 2)	−26	−78	−36		4.12
L rectal gyrus	0	38	−18	1443	5.38^**^
L middle orbital gyrus	−6	34	−12		5.24*
L anterior cingulate cortex, including vmPFC°	0	52	−2		4.82
L superior frontal gyrus	−20	22	62	681	4.77
L middle frontal gyrus	−26	28	56		4.67
L middle frontal gyrus	−24	24	46		4.49
L postcentral gyrus	−42	−20	48	289	4.93*
L middle temporal gyrus	−64	−22	−16	273	5.03*
R PCun	6	−50	54	2060	7.89^***^
L PCun	−8	−42	64		5.46^**^
R superior parietal lobule	16	−58	62		5.37^**^
R middle temporal gyrus	42	−66	0	3969	10.35^***^
R middle temporal gyrus, including pSTS°	42	−64	10		10.08^***^
R middle temporal gyrus, including TPJ°	56	−62	10		8.58^***^
L middle occipital gyrus, including pSTS°	−44	−68	4	2801	8.35^***^
L angular gyrus, including TPJ°	−48	−76	26		5.22*
L middle occipital gyrus	−12	−102	6		5.21*
R superior occipital gyrus	28	−82	38	196	4.83
ROI: dmPFC	0	54	44	42	4.17^**^
Inconsistent: Non-social Sequencing > Social Sequencing					
R precentral gyrus	36	−24	58	31 727	20.46^***^
L cerebellum (IV–V)	−20	−50	−24		20.16^***^
R precentral gyrus	42	−20	62		17.8^***^
R middle frontal gyrus	32	40	26	340	5.14*
L middle frontal gyrus	−34	36	28	749	6.24^***^
L middle frontal gyrus	−38	44	32		6.08^***^
L middle frontal gyrus	−44	36	32		5.85^**^
L rolandic operculum	−44	−4	10	4724	10.70^***^
L putamen	−28	−2	2		8.83^***^
L caudate nucleus	−18	−12	20		8.82^***^
L precentral gyrus	−34	−14	64	200	6.99^***^
L postcentral gyrus, including aIPS°	−42	−32	46	3784	12.79^***^
L postcentral gyrus	−60	−20	22		10.48^***^
L inferior parietal lobule	−58	−22	44		8.45^***^
Consistent: Social Sequencing > Non-social Sequencing					
R PCun	6	−50	54	893	6.79^***^
L PCun	−12	−58	60		4.52
R superior parietal lobule	16	−58	62		3.82
R middle temporal gyrus, including pSTS°	42	−64	14	2561	8.27^***^
R middle temporal gyrus	44	−64	0		7.26^***^
R middle temporal gyrus	52	−68	0		6.76^***^
L middle temporal gyrus, including pSTS°	−42	−68	8	1392	6.28^***^
L middle occipital gyrus	−36	−66	18		4.86
L middle temporal gyrus, including TPJ°	−44	−52	16		4.85
Consistent: Non-social Sequencing > Social Sequencing					
R precentral gyrus	36	−24	58	48 193	19.12^***^
L cerebellum (IV–V)	−18	−50	−24		19.02^***^
R precentral gyrus	42	−20	62		16.94^***^
R middle frontal gyrus	28	52	26	619	5.34^**^
R middle frontal gyrus	30	38	24		4.72
R superior frontal gyrus	26	44	14		3.43
L precentral gyrus	−34	−14	62	279	6.97^***^
L cerebellum lobule IX	−2	−38	−42	244	6.36^***^

*Note*: Coordinates refer to the MNI stereotaxic space. Whole-brain and ROI analysis threshold at voxel-wise uncorrected *P* < 0.001 with voxel extent > 10, with cluster-wise FWE corrected *P *< 0.05. Only the highest peaks of each cluster are shown, except for the cerebellum and significant ROIs. L = left, R = right.

* *P* < 0.05,

** *P* < 0.01,

*** *P* < 0.001 (peak FWE corrected) after whole-brain analysis. °*P* < 0.001 cluster-level FWE corrected using a small volume correction of a sphere with a 5- or 10-mm radius and centered around a priori MNI coordinate. These results are also summarized in Table 6.

**Table 6.** Overview of the ROIs and their activation

Functional domain	Social Sequencing		Social mentalizing				Action observation				Spatial memory
Function/ROI	Crus 2	Crus 1	PCun	vmPFC	dmPFC	TPJ	pSTS	PMC	aIPS	SMA	PHG
Observation Phase											
*Social vs Non-Social*											
Inconsistent: Social Sequencing > Non-Social Sequencing	✓	✓	✕	✓	✓	✓	✓	✕	✕	✕	✕
Inconsistent: Social Sequencing < Non-Social Sequencing	✕	✕	✕	✕	✕	✕	✓	✓	✓	✓	✓
Consistent: Social Sequencing > Non-Social Sequencing	✕	✕	✕	✕	✕	✓	✕	✕	✕	✕	✕
Consistent: Social Sequencing < Non-Social Sequencing	✕	✕	✓	✕	✕	✕	✕	✓	✓	✓	✓
*Sequencing vs Non-Sequencing*											
Inconsistent: Social Sequencing > Social Non-Sequencing	✕	✕	✕	✕	✕	✕	✕	✕	✕	✕	✓
Inconsistent: Social Sequencing < Social Non-Sequencing	✕	✕	✓	✕	✕	✕	✕	✓	✓	✓	✓
Consistent: Social Sequencing > Social Non-Sequencing	✕	✕	✕	✕	✕	✕	✕	✕	✕	✕	✕
Consistent: Social Sequencing < Social Non-Sequencing	✕	✕	✓	✕	✕	✓	✓	✓	✓	✓	✓
Reproduction Phase											
Inconsistent: Social Sequencing > Non-Social Sequencing	✓	✓	✓	✓	✓	✓	✓	✕	✕	✕	✕
Inconsistent: Social Sequencing < Non-Social Sequencing	✕	✕	✕	✕	✕	✕	✕	✓	✓	✓	✓
Consistent: Social Sequencing > Non-Social Sequencing	✕	✕	✓	✕	✕	✓	✓	✕	✕	✕	✕
Consistent: Social Sequencing < Non-social Sequencing	✕	✕	✕	✕	✕	✕	✕	✓	✓	✓	✓

*Note*: ROIs activated in different contrasts, using a radius of 5 mm for Cerebellar Crus 1 and 2 and 10 mm for mentalizing (PCun, vmPFC, dmPFC and TPJ), action observation networks (pSTS, PMC, aIPS and SMA) and Spatial bilateral PHG. Activation in ROI: ✓ = in at least one hemisphere, ✕ = in no hemisphere.

We hypothesized that both the observation and the reproduction of trajectories would potentially generate stronger cerebellar activation, in line with the sequence detection and generation role of the cerebellum (Braitenberg *et al.*, 1997; Heleven *et al.*, 2021). We therefore discuss the results of these two phases in succession.

### Observation phase: memorizing sequences of trajectories

#### Social Sequencing vs Non-social Sequencing

As noted above, we hypothesized that a Social > Non-social contrast would reveal activation in all mentalizing key areas, especially in the inconsistent condition, but not or less so in the action observation areas.

**Fig. 3.**
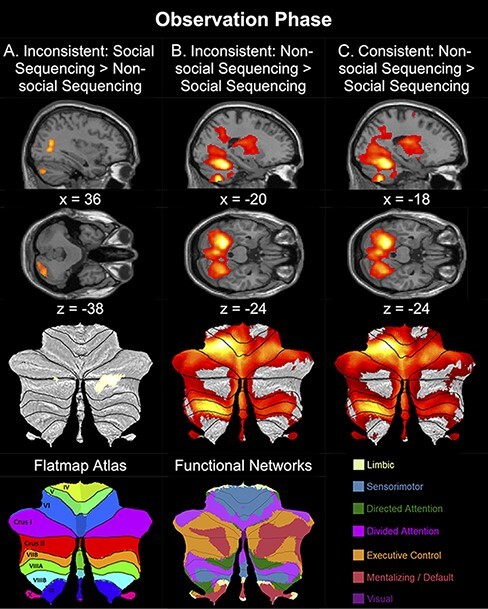
Top: Sagittal and transverse views of the contrasts during the Observation Phase are shown at an uncorrected threshold of *P* < 0.001. Middle: Activations in the posterior cerebellum of the same contrasts are shown on a SUIT flatmap (King *et al*., 2019) at an uncorrected threshold of *P* < 0.001. The contrasts between Social Sequencing *vs* Non-social Sequencing in the Inconsistent condition (A) strongly activate Crus 1 and 2 in the mentalizing network, whereas the other contrasts (B and C) show activation in other networks. Bottom: SUIT flatmap atlas showing the cerebellar lobules from King *et al*. (2019) and functional networks from Buckner *et al*. (2011). Warm colors (shades of orange and yellow) on flatmaps correspond to positive brain activation. Note that we only included contrasts where we found cerebellar activations.

In the Inconsistent condition, as hypothesized, the Social Sequencing > Non-social Sequencing contrast revealed significant ROI activation in the posterior cerebellum (Crus 1 and 2), the ventral mPFC (vmPFC), dorsal mPFC (dmPFC) and bilateral TPJ from the mentalizing network, and the bilateral pSTS from the action observation network (Table 3; Figure 3A). Additional whole-brain activation was revealed in the right rectal gyrus, left middle frontal gyrus, left paracentral lobule, bilateral middle temporal gyrus and right cuneus. The opposite contrast (Non-social Sequencing > Social Sequencing) revealed significant ROI activation in PMC, bilateral aIPS and bilateral SMA from the action observation network, and left PHG. Additional whole-brain activation was revealed in the left cerebellum (IV–V), right precentral gyrus, right middle frontal gyrus and left precentral gyrus (Table 3; Figure 3B).

In the Consistent condition, as hypothesized, the Social Sequencing > Non-social Sequencing contrast revealed less ROI activation: only significant ROI activation in the bilateral TPJ from the mentalizing network and bilateral pSTS from the action observation network. Additional whole-brain activation was revealed in the paracentral lobule extended to bilateral PCun and the bilateral middle temporal gyrus. The opposite contrast (Non-social Sequencing > Social Sequencing) revealed significant ROI activation in the PCun from the mentalizing network, the PMC, bilateral aIPS and bilateral SMA from the action observation network, and the bilateral PHG. Additional whole-brain activation was revealed in the left cerebellum (IV–V) and bilateral precentral gyrus (Table 3; Figure 3C).

In sum, in line with the hypothesis, the posterior cerebellar Crus 1 and 2, as well as the cortical mentalizing network, are predominantly active under the inconsistent social condition. In contrast, the action observation network appears to be underactive in both (in)consistent conditions, resulting in an unexpected stronger activation given the opposite Non-social Sequencing > Social Sequencing contrast.

#### Social sequencing vs social non-sequencing

We hypothesized that a Social Sequencing > Social Non-sequencing contrast would reveal activation in the posterior cerebellar Crus, while mentalizing and action observation areas are relatively insensitive to a sequencing manipulation.

In the Inconsistent condition, the Social Sequencing > Social Non-sequencing contrast revealed significant ROI activations in the bilateral PHG. Additional whole-brain activation was revealed in the bilateral PCun extended to the hippocampus. The opposite contrast (Social Non-sequencing > Social Sequencing) revealed significant ROI activations in the PCun, bilateral PMC, bilateral aIPS and bilateral SMA from the action observation network, and the bilateral PHG. Additional whole-brain activation was revealed in the left cerebellum (VI), right precentral gyrus and left middle temporal gyrus (Table 4). Note that although the bilateral PHG is observed in both directions of the ROI analysis, their exact location is not entirely identical (i.e. Social Sequencing > Social Non-sequencing: −34 −38 −8 and 34 −34 −6, which is somewhat more anterior and superior than Social Non-sequencing > Social Sequencing: −26 −44 −18 and 30 −40 −18).

In the Consistent condition, the Social Sequencing > Social Non-sequencing contrast revealed whole-brain activation in the bilateral PCun extended to the thalamus. The opposite contrast (Social Non-sequencing > Social Sequencing) revealed significant ROI activations in the PCun, bilateral TPJ from the mentalizing network, left pSTS, bilateral PMC, bilateral aIPS and bilateral SMA from the action observation network, and the bilateral PHG. Additional whole-brain activation was revealed in the right precentral gyrus extended to left cerebellum VI, right inferior frontal gyrus and right superior temporal gyrus (Table 4).

In sum, the Social Sequencing *vs* Social Non-sequencing results suggest that mentalizing and action observation ROIs were more active when identifying social sequences were not essential in the task (i.e. Non-sequencing conditions). Although we hypothesized that sequencing is not inherently associated with social cognition, we did predict it to be an essential function of the cerebellum and therefore would recruit more posterior cerebellar activation. This was not the case. Potential explanations could be that the movement of picking up the gift might have interfered with the smooth processing of the sequential movements of the protagonist, or that the inconsistency might have added a sequential component (e.g. a shift in the anticipated actions) even in the Non-sequencing control condition. To rule out this explanation, we reran the above sequencing analysis with the beginning of the trajectory as onset time (before participants were aware of any potential inconsistency) instead of setting the onset time at gift pick-up time. Again, contrary to our hypothesis, the results revealed largely similar results in our ROIs, again with no activation in Crus 1 or 2 in none of the sequencing *vs* non-sequencing contrasts (see Supplementary Material, Supplementary Tables S1 and S2).

### Reproduction phase: reproducing sequences of trajectories

#### Social Sequencing vs Non-social Sequencing

**Fig. 4.**
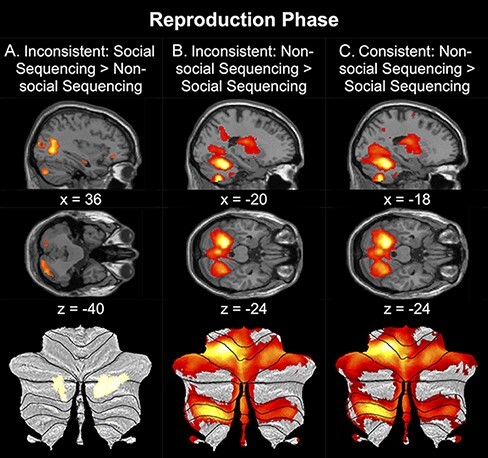
Top: Sagittal and Transverse views of the contrasts during the Reproduction Phase are shown at an uncorrected threshold of *P* < 0.001. Bottom: Activation in the posterior cerebellum of the same contrasts shown on a SUIT flatmap (King *et al*., 2019) at an uncorrected threshold of *P* < 0.001. The contrasts between Social Sequencing *vs* Non-social Sequencing in Inconsistent condition (A) strongly activate Crus 1 and 2 in the mentalizing network, whereas the other contrasts (B and C) show activation in other networks. Note that we only included contrasts where we found cerebellar activations.

In the Inconsistent condition, similar to the Observation Phase, the Social Sequencing > Non-social Sequencing contrast revealed significant ROI activation in posterior cerebellar activation (Crus 1 and 2), the PCun, vmPFC, dmPFC, bilateral TPJ from the mentalizing network and the bilateral pSTS from the action observation network (Table 5; Figure 4A). Additional whole-brain activation was revealed in the left rectal gyrus, left superior frontal gyrus, left postcentral gyrus, bilateral middle temporal gyrus, bilateral PCun, left middle occipital gyrus and right superior occipital gyrus. The opposite contrast (Non-social Sequencing > Social Sequencing) revealed significant ROI activation in the left PMC, bilateral aIPS and bilateral SMA from the action observation network, and the left PHG. Additional whole-brain activation was revealed in the left cerebellum (IV–V), bilateral precentral gyrus, bilateral middle frontal gyrus, left rolandic operculum and left postcentral gyrus (Table 5; Figure 4B).

In the Consistent condition, the Social Sequencing > Non-social Sequencing contrast revealed less ROI activation: only significant ROI activation in the PCun and bilateral TPJ from the mentalizing network, and in the bilateral pSTS from the action observation network. Additional whole-brain activation was revealed in the bilateral middle temporal gyrus. The opposite contrast (Non-social Sequencing > Social Sequencing) revealed significant ROI activation in the left PMC, bilateral aIPS and bilateral SMA from the action observation network, and left PHG. Additional whole-brain activation was revealed in the bilateral precentral gyrus extending to the left cerebellum (IV–V) and right middle frontal gyrus (Table 5; Figure 4C).

In sum, these results are largely consistent with the observation phase. Again, in line with the hypothesis, the posterior cerebellar Crus 1 and 2, as well as the cortical mentalizing network, are predominantly active under inconsistent conditions. And again, the action observation network shows stronger activation given the opposite Non-social Sequencing > Social Sequencing contrast in both (in)consistent conditions.

## Discussion

In this study, we examined the violation of dynamic sequences of expected social outcomes that are pervasive in human interaction and navigation, such as offering a gift. We hypothesized that the social posterior cerebellar Crus (1 and 2) might play a critical role in detecting the discrepancy between expectation and outcomes, apart from other cortical areas involved in mentalizing and spatial navigation. As expected, our results provide compelling evidence that the posterior cerebellar Crus supports detecting violations in dynamic social scenarios. This extends the role of the cerebellum from ‘detecting movement error’ to ‘detecting social expectation violations’ in interactive social navigation.

### The cerebellum in detecting violations in social navigation

We tested our social violation hypothesis of the cerebellum by modifying an earlier paradigm involving dynamic social navigation with a novel expectation violation manipulation. By moving through a spatial grid, protagonists (i.e. smurfs) offered gifts to another agent that were desired or not. As hypothesized, activation in the right cerebellar Crus 1 and 2 increased upon detecting violations (i.e. picking up an undesired gift) while learning sequences through the grid in social interactions (*vs* non-social sequences). The same pattern of results was found when reproducing the sequences, confirming the cerebellar function in sequence generation (Molinari *et al.*, 2008).

#### Social interaction boosts cerebellar activation

Our findings document and extend the social function of the cerebellum in a social navigation task. While our previous research using a similar goal-directed trajectory paradigm with a single protagonist revealed activation in the left Crus 1 (Li *et al.*, 2021), the current task involving an interaction between two protagonists revealed larger right Crus 1 and 2 activation under social inconsistent conditions. Hence, rendering the situation more socially engaging and, in particular, socially unacceptable seemed to have increased the contribution of the posterior cerebellum. This is consistent with prior research documenting that generating the correct sequence of actions in higher level social contexts (i.e. while inferring others’ social beliefs) has more detrimental effects on cerebellar patients (Van Overwalle *et al.*, 2019a) and recruits more posterior cerebellar Crus activation than contexts involving overtrained social scripts (e.g. shopping) or physical events (e.g. car accident) (Heleven *et al.*, 2019).

#### Social inconsistencies also recruit the cerebellum

The present findings point to the role of the cerebellum in inconsistent social interaction and navigation, when social behaviors are inconsistent with norms and expectations (as opposed to inconsistencies in non-social events). This supports earlier research on the role of the posterior cerebellum in disruptions of sequences of social trajectories (Li *et al.*, 2021) and extends it to inconsistencies at the social level only. This aligns well with the general role of the cerebellum in signaling errors, both in social sequences and in the social implications of actions (Van Overwalle *et al.*, 2019b). As noted earlier, a recent study on trait-implying behaviors (Pu *et al.*, 2021) also demonstrated that the posterior cerebellum detected violations at the social level, such as actions that violate (*vs* confirm) expectations based on the traits of human agents. Even during implicit sequence learning, the posterior cerebellum was involved when implicitly learning social sequences and detecting random violations in this sequence (Ma *et al.*, 2021).

It is important to note, however, that in a consistent sequencing context, the posterior cerebellum was not activated in the social *vs* non-social comparison. One potential explanation for this unexpected finding relates to the importance and relevance of sequencing. The inconsistency manipulation may have become so salient that it received processing precedence over other information so that even in the non-sequencing control condition, participants may have paid relatively great attention to the sequences preceding or following the inconsistency, potentially also in an attempt to explain the inconsistency. Consequently, other distinctions in the manipulation, such as human *vs* non-human agents, became less relevant or salient. A more intriguing explanation suggests a qualification of the original social sequencing hypothesis of the social cerebellum put forward by (Van Overwalle *et al.* 2019b). Perhaps not so much representing upcoming action sequences by self and others, but rather monitoring one’s alignment with others’ actions and thoughts, and their timing, is more critical (for a similar view, see Deschrijver and Palmer, 2020). Both strategies—monitoring the sequences of others’ social actions or misalignment with others’ sequences—align with the overall idea of the sequence detection hypothesis (Leggio and Molinari, 2015) but place a somewhat different emphasis on the critical locus of the process. Despite these differences, these ideas converge on the consensual notion that the cerebellum compares novel information with expectations based on internal representations. If the prediction is upheld, cerebellar feedback will prepare related cortical areas for the incoming stimulus so that it can be perceived and reacted upon more effectively. In contrast, when events are unexpected, a cerebellar error signal will accelerate the processing of unanticipated inputs and prepare behavioral responses in reaction to new possibilities.

#### A cerebellar role in sequencing?

The comparison between Social Sequencing and Social Non-sequencing in this study failed to highlight the role of the posterior cerebellum in sequencing. When participants were required to memorize the sequence of the protagonist as opposed to simply observing the sequence, the posterior cerebellar Crus was not activated. This contradicts our previous study on social trajectories of single protagonists (Li *et al.*, 2021), where an effect of memorizing sequencing *vs* not memorizing them was observed on cerebellar activation. There might be several potential explanations for this unexpected result. One theoretical explanation, put forward earlier, is that the social violation became so salient or dominant that it received processing precedence over other information. It is likely that this may have also directed the participants’ attention to the sequence directly preceding or following the inconsistency even in the Social Non-sequencing condition, in an attempt to explain the inconsistency, so that any differences with the Social Sequencing condition were minimized. A similar conclusion follows from the misalignment argument discussed earlier (Deschrijver and Palmer, 2020). When actions conflict with another agent, monitoring their exact order in the cerebellum becomes much less relevant as opposed to monitoring their social consequences. A methodological explanation is that the Social Non-sequencing (i.e. observation only) control condition always came at the end of the experiment. After being trained to focus on sequences during the preceding experimental phase, participants may have continued to do so to some degree spontaneously, thereby obliterating major distinctions between the Sequencing and Non-sequencing conditions. Another methodological explanation is the critical timepoint for the fMRI analysis, that is, the gift pick-up time in both Sequencing and Non-sequencing conditions. However, an additional analysis where we analyzed the effects of sequencing at the start of the trajectory before participants were aware of any potential violation did not change the results. Note that this does not rule out the prior theoretical arguments, as participants might have been constantly on the lookout for inconsistencies in the social behavior (as 40% of the trials were inconsistent). Future research is needed to unravel the contribution of these intriguing theoretical and methodological factors.

To sum up, in agreement with the functional role of the posterior cerebellar Crus in social sequence learning, our results substantiate its inherent role of sequencing for social cognition, especially when a protagonist moves to an unexpected goal that violates the social norms and expectations of the observer.

### Cerebral cortex in detecting violations in social navigation

In addition to the contribution of the cerebellum, we found that cortical areas related to the processing of social trajectories and social violations were also involved, including mentalizing and action observation processes.

#### Mentalizing processes are boosted under social (in)consistent conditions

We found that the key cortical regions of the mentalizing network, including the mPFC and TPJ, were involved in detecting social prediction errors, much in line with earlier research (Ma *et al.*, 2012; Wang *et al.*, 2021). However, consistent with our hypothesis, note that the TPJ was strongly activated regardless of consistent or inconsistent social expectations. This confirms the robustness of our social mentalizing manipulation in the present social trajectory paradigm.

The role of the TPJ is in line with previous findings that this area is essential for processing temporary goals, intentions and beliefs of others (Abu-Akel *et al.*, 2020; Guterstam *et al.*, 2021; Ma *et al.*, 2021; Schuwerk *et al.*, 2021). A nearby area, the pSTS, which belongs to the action observation system, was robustly activated in Social Sequencing conditions as well. This finding is consistent with previous research demonstrating its role in social interaction, such as observing intentional actions (Saxe *et al.*, 2004; Arioli *et al.*, 2018; Yan *et al.*, 2020). Recently, Arioli *et al.* (2021) also found that the pSTS was recruited during social interactions, particularly by negative ones, such as competitive and conflicting social stimuli. Most importantly, the TPJ and pSTS were simultaneously activated with the posterior cerebellum in dynamic social sequences when detecting violations, which is in accordance with functional and structural loops between the bilateral posterior cerebellum with the bilateral TPJ (Van Overwalle *et al.*, 2020b) and the STS (Sokolov *et al.*, 2012, 2014).

Notably, the pSTS, which is a deemed part of the action observation network (Castelli *et al.*, 2000; Arioli *et al.*, 2018), was always activated together with the key mentalizing TPJ. However, this is in accordance with previous studies that demonstrate that the pSTS is highly sensitive to whole-body motion (see meta-analysis by Van Overwalle and Baetens, 2009), which was schematically represented in our study, and responds to animated goal-directed movements more than movements that are not perceived as goal-directed (Gao *et al.*, 2012).

The same pattern of TPJ results was found for the PCun. A whole-brain analysis unveiled PCun activation in Social Sequencing (*vs* Non-social and Non-sequencing conditions) given both consistent and inconsistent conditions. This supports the view that the PCun is linked to learning social navigational sequences (Van Overwalle and Baetens, 2009; Schurz *et al.*, 2014).

The mPFC, another key mentalizing area, was activated only when inconsistencies arose in Social Sequencing. This area has been widely acknowledged as one of the central regions in the mentalizing network (Van Overwalle, 2009; Baetens *et al.*, 2017). In research on social sequencing, this area was strongly activated when observers made social inferences on protagonists’ traits (Pu *et al.*, 2021). In our study, such trait inferences were most likely when protagonists violated social norms by presenting undesired gifts.

#### Action observation processes are diminished under inconsistent conditions

With respect to action observation, all major areas (PMC, aIPS and SMA) were involved in Non-social Sequencing (*vs* Social Sequencing) conditions regardless of consistency, except for the pSTS as discussed above. This finding may appear surprising. However, note that we hypothesized that action observation would probably play a diminished role, as social inconsistencies predominantly recruit the mentalizing network (Van Overwalle and Baetens, 2009; Morrison *et al.*, 2012; Aridan and Mukamel, 2016). What we had not expected is that its role would be so marginal that opposite activation patterns would ensue, with relatively greater activation in the non-social as opposed to social conditions. An additional explanation might be that the action observation network is preferentially sensitive to realistic biological movement. The schematic step-by-step movements in our design, which are not biologically realistic, may have, therefore, dampened rather than promoted action observation processes in the social conditions. Additional evidence is needed to demonstrate whether this is a robust result and which of our interpretations is most valid.

#### (Para)hippocampus and non-social object memorizing

The PHG, surrounding the hippocampus, was activated in Non-social as opposed to Social Sequencing conditions, illustrating its importance in encoding object locations and retrieving spatial memory (Kühn and Gallinat, 2014). This finding is consistent with previous findings that PHG activation was more pronounced during reading sentences about inanimate objects over social and social-emotional actions (Mellem *et al.*, 2016).

It is important to note that most patterns discussed above concerning the observation phase of the experiment were also revealed during the reproduction phase of the trajectory.

### Limitations

One limitation of the present study is the lack of a counterbalanced design. We only presented social after non-social conditions to avoid possible anthropomorphizing of non-social pen or ink, something that humans are readily inclined to do when observing moving objects (cf., Heider–Simmel animations; Heider and Simmel, 1944; Schafroth *et al.*, 2021). However, an order effect cannot be avoided and possible systematic variations might have been introduced. Another limitation is that we only included the negative aspect of inconsistency. Violation of expectations can cause either positive or negative outcomes, raising both unexpectedly happy and sad consequences. It will be interesting to determine whether expectation violation is encoded in the cerebellum only when undesired (and thus requiring corrective action) or also desired, and whether these violations also involve the cerebral cortex. Reward resulting from outcomes, which will influence the recipient’s reaction to the violation, can be another critical factor to include. We note these points as limitations of the current work and an interesting line for future research.

## Conclusions

Every divergent thought is different in its own way and can influence your well-being in distinct ways. Therefore, it may be more important to understand how and when others think differently than to infer similar ideas held by the majority. This study documented the critical role of the posterior cerebellum in detecting expectation violations in dynamic social interactions using a social–spatial navigation paradigm, that is, when a protagonist moved to an unexpected goal that violated social norms and expectations. The present findings deepen our understanding of the role of the cerebellum in predictive social processing and suggest that the notion of action sequences represented in cerebellar internal models may be extended to the social navigation domain. Our findings also demonstrate the importance of two critical issues: (i) incorporating dynamic social interactive contexts to better understand the neural substrates subserving everyday social cognition and (ii) the utility of examining cerebellar activation in social cognitive processes. The social navigation paradigm employed here allows us to link specific social characteristics to brain activity in the real world, exhibiting potential implications for the diagnosis and treatment of impairments in dynamic social functioning.

## Supplementary Material

nsac047_Supp

## Data Availability

All requested (pseudonymized or anonymous) data are available upon request to M.L. at meijia.li@vub.be, excluding data that allow identifying individual participants. If relevant, all manuals and code for processing the data are also available together with the data.
