# Supplementary material for: Mind your step: social cerebellum in interactive navigation
Source: Soc Cogn Affect Neurosci. 2022 Jul 22;18(1):nsac047. doi: 10.1093/scan/nsac047 (PMC9949501; doi:10.1093/scan/nsac047)
Supplement: nsac047_Supp [file nsac047_supp.zip › Supplementary Material.docx]

Mind Your Step: Social Cerebellum in Interactive Navigation

**Supplementary Materials**

Table S1. Whole-brain and ROI analysis during the observation of the trajectories for Social Sequencing and Social Non-sequencing control contrasts (Onset = the beginning of the trajectory).

|  | Contrasts and Anatomical Label | MNI coordinate | | | Voxels | max t |
| --- | --- | --- | --- | --- | --- | --- |
|  |  | *x* | *y* | *z* |  |  |
| **Inconsistent: Social Sequencing > Social Non-sequencing** | | | |  |  |  |
|  | L MCC | -2 | -26 | 48 | 480 | 4.71 |
|  | R Hippocampus | 36 | -32 | -6 | 12513 | 8.4*** |
|  | R Hippocampus | 34 | -42 | 4 |  | 8.11*** |
|  | L Hippocampus | -36 | -40 | -6 |  | 7.86*** |
|  | L Postcentral Gyrus | -22 | -40 | 68 | 671 | 4.61 |
|  | L Postcentral Gyrus | -16 | -40 | 76 |  | 4.53 |
|  | L Postcentral Gyrus | -24 | -26 | 74 |  | 3.97 |
|  | R Superior Occipital Gyrus | 16 | -92 | 30 | 276 | 4.76 |
| **Inconsistent: Social Non-sequencing > Social Sequencing** | | | |  |  |  |
|  | L Cerebellum (IV-V) | -20 | -50 | -24 | 12185 | 10.94*** |
|  | L Superior Parietal Lobule | -28 | -58 | 54 |  | 10.28*** |
|  | L Inferior Parietal Lobule, including aIPS° | -46 | -36 | 42 |  | 10.21*** |
|  | R Insula Lobe | 32 | 26 | 0 | 201 | 5.52** |
|  | L Insula Lobe | -30 | 24 | 4 | 257 | 5.17* |
|  | R Precentral Gyrus, including PMC° | 54 | 4 | 34 | 452 | 7.21*** |
|  | R Putamen | 28 | -4 | 2 | 399 | 6.37*** |
|  | L Pallidum | -24 | -12 | 4 | 188 | 4.37 |
|  | R Precentral Gyrus | 36 | -22 | 56 | 13893 | 14.26*** |
|  | R Superior Frontal Gyrus | 28 | -8 | 62 |  | 10.6*** |
|  | R Posterior-Medial Frontal, including SMA° | 6 | 0 | 56 |  | 10.15*** |
|  | Thalamus: Somatosensory | -4 | -22 | -6 | 815 | 6.59*** |
|  | Thalamus: Prefrontal | 4 | -20 | -6 |  | 6.34*** |
|  | R Thalamus | 14 | -20 | 8 |  | 5.89** |
| **Consistent: Social Sequencing > Social Non-sequencing** | | | |  |  |  |
|  | L Caudate Nucleus | -16 | 22 | 14 | 6438 | 7.05*** |
|  | L Parahippocampal Gyrus | -36 | -40 | -4 |  | 6.95*** |
|  | L Precuneus, including PHG° | -26 | -52 | 14 |  | 6.93*** |
|  | R Precuneus | 34 | -46 | 6 | 2624 | 8.58*** |
|  | R Hippocampus | 36 | -34 | -4 |  | 7.05*** |
|  | R Hippocampus | 38 | -26 | -12 |  | 6.33*** |
| **Consistent: Social Non-sequencing > Social Sequencing** | | | |  |  |  |
|  | R Precentral Gyrus | 36 | -24 | 58 | 34247 | 14.24*** |
|  | L Cerebellum (IV-V) | -18 | -50 | -24 |  | 12.04*** |
|  | R Superior Parietal Lobule | 18 | -64 | 54 |  | 11.11*** |
|  | R Insula Lobe | 32 | 26 | -4 | 311 | 6.24*** |
|  | L Insula Lobe | -30 | 24 | 4 | 314 | 5.59** |
|  | R Precentral Gyrus, including PMC° | 54 | 4 | 34 | 620 | 6.65*** |
|  | R Middle Frontal Gyrus | 46 | 24 | 32 |  | 4.39 |
|  | R IFG (p. Opercularis) | 36 | 12 | 30 |  | 4.31 |
|  | R Putamen | 30 | -2 | 0 | 482 | 6.56*** |
|  | L Pallidum | -22 | -8 | 4 | 170 | 5.52** |
|  | Thalamus: Somatosensory | -4 | -24 | -6 | 997 | 6.43*** |
|  | Thalamus: Premotor | 8 | -18 | -6 |  | 6.31*** |
|  | R Thalamus | 16 | -22 | 10 |  | 6.2*** |
|  | Lobule IX | -2 | -38 | -42 | 196 | 5.12* |
| **Social Sequencing > Non-sequencing** | |  |  |  |  |  |
|  | L Posterior-Medial Frontal | -6 | -20 | 60 | 527 | 5.18* |
|  | L MCC | -2 | -26 | 48 |  | 4.6 |
|  | L MCC | 0 | -8 | 38 |  | 4.5 |
|  | L Superior Parietal Lobule | -20 | -40 | 62 | 699 | 4.66 |
|  | L Postcentral Gyrus | -24 | -26 | 74 |  | 4.34 |
|  | L Paracentral Lobule | -10 | -18 | 78 |  | 4.32 |
|  | R Precuneus | 34 | -46 | 6 | 13464 | 9.45*** |
|  | R Hippocampus | 36 | -32 | -6 |  | 8.71*** |
|  | L Precuneus, including PHG° | -26 | -52 | 14 |  | 8.33*** |
|  | L Cuneus | 0 | -88 | 30 | 204 | 4.74 |
| **Non-sequencing > Social Sequencing** | |  |  |  |  |  |
|  | R Precentral Gyrus, including aIPS° | 36 | -24 | 58 | 34181 | 16.18*** |
|  | L Cerebellum (IV-V) | -20 | -50 | -24 |  | 12.95*** |
|  | L Superior Parietal Lobule | -28 | -58 | 54 |  | 12.14*** |
|  | R Insula Lobe | 32 | 26 | -4 | 317 | 6.65*** |
|  | L Insula Lobe | -30 | 24 | 4 | 361 | 6.11** |
|  | R Precentral Gyrus, including PMC° | 54 | 4 | 34 | 856 | 7.9*** |
|  | R Precentral Gyrus | 58 | 8 | 16 |  | 4.96 |
|  | R Middle Frontal Gyrus | 46 | 24 | 34 |  | 4.74 |
|  | R Putamen | 28 | -4 | 2 | 530 | 7.29*** |
|  | L Pallidum | -22 | -8 | 4 | 245 | 5.44** |
|  | Thalamus: Premotor | -4 | -22 | -6 | 1150 | 7.41*** |
|  | Thalamus: Prefrontal | 6 | -20 | -6 |  | 7.03*** |
|  | R Thalamus | 16 | -20 | 8 |  | 6.76*** |

*Note*. Coordinates refer to the MNI (Montreal Neurological Institute) stereotaxic space. Whole-brain and ROI analysis threshold at voxel-wise uncorrected p < 0.001 with voxel extent ≥ 10, with cluster-wise FWE corrected p < 0.05. Only the highest peaks of each cluster are shown, except for the cerebellum and significant ROIs. L = left, R = right. ^*^*p* < 0.05, ^**^*p* < 0.01, ^***^*p* < 0.001 (peak FWE corrected). ß° *p* < 0.001 cluster-level FWE corrected using a small volume correction of a sphere with 5- or 10-mm radius and centered around a priori MNI coordinate. These results are also summarized in Table S2. PMC = Premotor Cortex, aIPS = Anterior Intraparietal Sulcus, SMA = Supplementary Motor Area, PHG = Parahippocampal Gyrus.

Table S2. Overview of the regions of interest and their activation as predicted by their functionality (Onset = the beginning of the trajectory).

| Functional Domain | Social Sequencing | |  | Social Mentalizing | | | |  | Action Observation | | | |  | Spatial Memory |
| --- | --- | --- | --- | --- | --- | --- | --- | --- | --- | --- | --- | --- | --- | --- |
| Function / Region of interest (ROI) | Crus 2 | Crus 1 |  | PCun | vmPFC | dmPFC | TPJ |  | pSTS | PMC | aIPS | SMA |  | PHG |
| **Observation phase** |  |  |  |  |  |  |  |  |  |  |  |  |  |  |
| *Sequencing vs. Non-sequencing* |  |  |  |  |  |  |  |  |  |  |  |  |  |  |
| Social Sequencing > Social Non-sequencing | ✕ | ✕ |  | ✕ | ✓ | ✕ | ✕ |  | ✕ | ✕ | ✕ | ✕ |  | ✓ |
| Social Sequencing < Social Non-sequencing | ✕ | ✕ |  | ✓ | ✕ | ✕ | ✕ |  | ✓ | ✓ | ✓ | ✓ |  | ✕ |
| Consistent: Social Sequencing > Social Non-sequencing | ✕ | ✕ |  | ✕ | ✓ | ✕ | ✕ |  | ✕ | ✕ | ✕ | ✕ |  | ✓ |
| Consistent: Social Sequencing < Social Non-sequencing | ✕ | ✕ |  | ✓ | ✕ | ✕ | ✕ |  | ✓ | ✓ | ✓ | ✓ |  | ✕ |
| Inconsistent: Social Sequencing > Social Non-sequencing | ✕ | ✕ |  | ✕ | ✓ | ✕ | ✕ |  | ✓ | ✕ | ✕ | ✕ |  | ✓ |
| Inconsistent: Social Sequencing < Social Non-sequencing | ✕ | ✕ |  | ✕ | ✕ | ✕ | ✕ |  | ✓ | ✓ | ✓ | ✓ |  | ✕ |

*Note*. ROIs activated in different contrasts, using a radius of 5 mm for Cerebellar Crus 1 & 2, and 10 mm for mentalizing (PCun, vmPFC, dmPFC, TPJ), action observation networks (pSTS, PMC, aIPS, SMA) and Spatial bilateral PHG. Activation in ROI: ✓ = in at least one hemisphere, ✕ = in no hemisphere. PCun = Precuneus, vmPFC = Ventromedial Prefrontal Cortex, dmPFC = Dorsal Medial Prefrontal Cortex, TPJ = Temporo-Parietal Junction, pSTS = Posterior Superior Temporal Sulcus, PMC = Premotor Cortex, aIPS = Anterior Intraparietal Sulcus, SMA = Supplementary Motor Area, PHG = Parahippocampal Gyrus
